# Biomimetic Water Oxidation Catalyzed by a Binuclear Ruthenium (IV) Nitrido-Chloride Complex with Lithium Counter-Cations

**DOI:** 10.3390/biomimetics5010003

**Published:** 2020-01-16

**Authors:** Zinaida M. Dzhabieva, Gennady V. Shilov, Lidia V. Avdeeva, Vladislav V. Dobrygin, Virineya Yu. Tkachenko, Taimuraz S. Dzhabiev

**Affiliations:** 1 Institute of Problems of Chemical Physics of RAS, Moscow Region, Academician Semenov Ave., 1, 142432 Chernogolovka, Russia; djabieva.zinaida@yandex.ru (Z.M.D.); genshil@icp.ac.ru (G.V.S.);; 2 Faculty of fundamental physical and chemical engineering, Lomonosov Moscow State University, GSP-1, 1 Leninskiye Gory, 119991 Moscow, Russia; mr_phosgene@mail.ru

**Keywords:** biomimetic catalysis, water oxidation, oxygen, binuclear nitrido complex Ru(IV) with lithium counter-cations

## Abstract

The lithium salt of the binuclear nitrido complex of ruthenium (IV) Li_3_(Ru_2_NCl_8_·2H_2_O) was synthesized. Using UV spectroscopy and voltammetry, we studied complex behavior in aqueous solutions. It was found that in dilute solutions of this compound, Cl^−^ ions are replaced by H_2_O molecules, and the intra-sphere redox reaction between Ru (IV) and H_2_O, as well as the oxidation of water with the formation of oxygen and the acidic dissociation of coordinated water molecules also have been taking place. It was established by IR spectroscopy and ESI mass spectrometric analysis that not only the binuclear structure of the complex is preserved in acidic solutions, but also its dimerization product into the tetra-ruthenium dinitrido cluster Ru_4_N_2_O_5_^+^, which is a catalyst for the water oxidation reaction. The activity of the catalyst was TOF = 0.33 s^−1^, TON = 304.

## 1. Introduction

**Figure 1:**
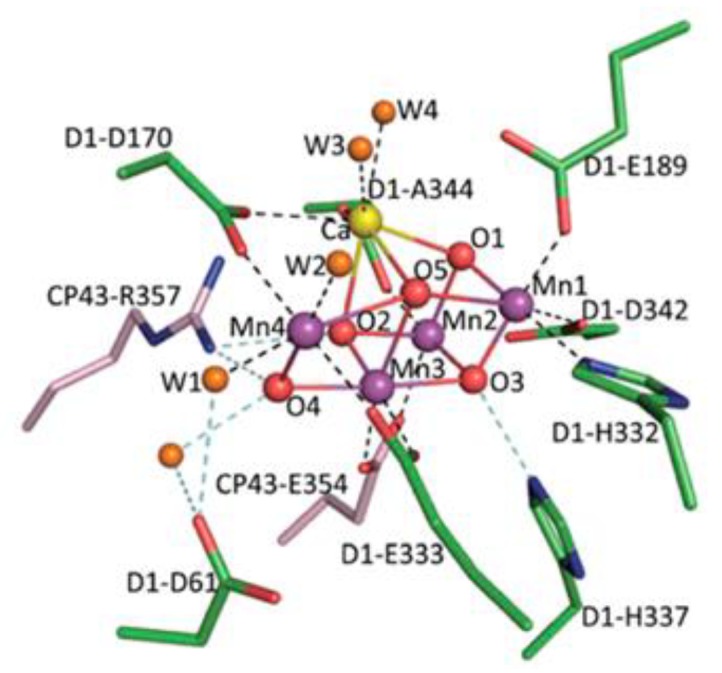
Structure of the manganese calcium cubane Mn_4_Ca and its ligand environment [1].

Green plants, algae, and some bacteria use water as a source of electrons in the process of photosynthesis to restore carbon dioxide into carbohydrates, resulting in the release of molecular oxygen. Water oxidation occurs in the coordination sphere of the manganese cofactor, which is associated with the polypeptide D1 of photosystem II (PSII), which according to the latest spectroscopic studies contains 4 Mn atoms and one calcium atom and has a cubane-like structure (Figure 1) [1].

In the oxygenic photosynthesis (OPS) in the active center of PS II, four single-electronic oxidants-cation-radicals P680^+●^ oxidize water according the reaction

4 P680^+^ + 2 H_2_O → 4 P680^+•^ + O_2_ + 4 H^+^. (1)

Modeling the natural process of water oxidation (artificial photosynthesis (APS)) is one of the effective ways of storing solar energy [2,3]. The APS for photoinduced water decomposition uses the principles of chemical reactions involved in the OPS. Of course, the most adequate functional chemical models of PSII are Mn^IV^_2m_ clusters [2,4,5]. However, in the biomimetic utilization of solar energy by means of photoinduced water decomposition, it is possible to use complexes of other transition metals: Co, Fe, Ru, Ir, etc. In search of biomimetic catalysts, ruthenium analogues of Mn-co play an outstanding role because of the wide range of possible states of ruthenium oxidation, which allows for the implementation of some processes of transformation of substrates into reaction products that are unique in their mechanism and selectivity. Currently, more than 200 ruthenium complexes are known as water oxidation catalysts in the AP. Often, such catalysts have a small number of revolutions, and the total yield of O_2_ in such systems is small [6,7,8,9,10]. This is due to a number of disadvantages of such complexes: Low activity due to the presence of organic ligands in the complexes, which are oxidized faster than water; and low stability, due to the presence of labile bond Ru-O-Ru. The main directions of research in world science are connected with the variation of the ligand environment of different transition metals.

At present, quad-core complexes Ru with polyoxometallic (POM) ligands are of great interest. Ruthenium complexes were discovered several years ago and differed only by the nature of X cations surrounding the polyanion ((Ru_4_(μ-O)_4_(μ-OH)_2_(H_2_O)_4_)·(γ-SiW_10_O_36_)_2_)^10−^ [11,12]. These catalysts were immediately perceived by the scientific community as an important element in solving the problems of water oxidation in the APS [13,14,15,16,17,18,19]. The interest to them is connected with the fact that the volumetric nucleophilic POM ligands surrounding the adamantane-like core of ruthenium Ru_4_O_6_ or the quasi-linear core of 4 Co, protect the labile bonds Me-O-Me, preventing the complexes to collapse in the harsh conditions of water oxidation reactions. POM ligands are chemical inorganic models of the protein part of the holoenzyme. In addition to POM ligands, the counter-cations (K^+^, Rb^+^, Cs^+^, Li^+^, etc.) surrounding the polyanionic nucleus also play a significant role in the activity and stability of catalysts. In early works, the structure and physical properties of the potassium salt of the binuclear nitrido complex were studied [20,21,22], which showed the possibility of synthesizing such a complex with lithium counter-cations, but no information was found on its use in the water oxidation reaction. Currently, only three works devoted to the water oxidation by Ce(IV) compounds catalyzed by ruthenium complexes with Li^+^ counter-cations have been published. These complexes in an acidic environment are more active than similar complexes with other counter-cations [11,23,24].

Therefore, various approaches have been studied to increase the activity and stability of ruthenium catalysts:

1. Replacement of organic ligands in complexes with inorganic or POM ligands;
2. Replacement of K^+^, Rb^+^, and Cs^+^ cations with the Li^+^ counter-cation;
3. Replacement of the oxygen bridge between ruthenium nuclei with a nitrogen bridge.

Thus, the aim of the study is to implement the proposed approaches and to create an effective stable inorganic ruthenium catalyst of water oxidation in the coordination sphere of the tetra-nuclear ruthenium (IV) nitrido-chloride complex with Li+ counter-cations, to characterize the complex by physico-chemical methods of analysis (X-ray diffraction analysis, IR and electronic spectroscopy, polarography, cyclic voltammetry, ESI mass spectrometric analysis and the kinetic method of investigation of the reaction of biomimetic water oxidation by Ce(IV) in the presence of binuclear complexes of ruthenium with N- and O-bridges, as well as assessment of their activity).

## 2. Materials and Methods

As a functional model of the water enzyme in the OPS (i.e., enzyme: water:plastochinon—oxidoreductase), the binuclear nitrido chloride ruthenium complex Li_3_(Ru_2_(μ-N)Cl_8_∙2H_2_O) was synthesized. Synthesis was performed using the method in Reference [25]. All operations were performed in air using commercial reagents (Fluka). The synthesis scheme is presented below.

### 2.1. Synthesis Binuclear Ruthenium (IV) Nitrido Chloride Complex with Lithium Counter-Cations

#### 2.1.1. Production of Pentachloronitrosilruthenate Li_2_(RuNOCl_5_)∙2H_2_O

2RuCl_3_ + 6LiNO_2_ + 6HCl → 2RuNOCl_3_ + 6LiCl + NO↑ + 3NO_2_ + 3H_2_O
2RuNOCl_3_ + 5LiNO_2_ $\overset{H_{2}O}{\to }$ Li_2_(RuNO(NO_2_)_4_OH) + 3LiCl + HNO_2_
Li_2_(RuNO(NO_2_)_4_OH) $\overset{\mathrm{HCl}}{\to }$ Li_2_(RuNOCl_5_)∙2H_2_O

LiNO_2_ (0.03471 M) was added to the warm solution of RuCl_3_ (0.01157 M) and 12.9 M HCl was added with constant mixing. After NO and NO_2_ were extracted, LiNO_2_ (0.02892 M) was added and HCl (12.9 M) was added, so the reaction was 6.45 M HCl. The reaction was for 3 h at *t* = 50 °C. The brown solution became raspberry. After the reaction was completed, the solution evaporated at room temperature to a small volume. Then the crystals were filtered and recrystallized from 6 M HCl. The yield: 1.7 g (70%). Found, %: CI—44.97; N—3.55; Ru—25.64; and Li—3.52 Calculated for H_4_Cl_8_Li_3_NRu_2_O_2_, %: Cl—45.00; N—3.53; Ru—25.61; and Li—3.55. IR spectrum, ν^as^ in cm^−1^: Ru-Cl 189, 285, 328; Ru-N-O 340; Ru-N 583; N-O 900.

#### 2.1.2. Obtaining the Binuclear Nitric Chlorido Ruthenium Complex Li_3_(Ru_2_(μ-N)Cl_8_∙2H_2_O

Li_2_(RuNOCl_5_)∙2H_2_O $\overset{{\mathrm{SnCl}}_{2}\cdot \mathrm{HCl}}{\to }$ Li_3_(Ru_2_(μ-N)Cl_8_∙2H_2_O

In addition to the freshly prepared Li_2_(RuNOCl_5_) solution (0.00826 M), SnCl_2_ solution was added (0.0219 M SnCl_2_∙2H_2_O in 20% HCl). The mixture was heated when mixed for 3 h and *t* = 80–90 °C. The raspberry solution became red-brown. The solution was quickly cooled down in a bath (alcohol mixed with liquid nitrogen). The dropped crystals were filtered out. The substance was washed with a minimum amount of cold distilled water and then with an acetone solution (35%) and recrystallized from 3 M HCl. The yield: 1.8 g (50%). Found, %: Cl—50.93; N—2.56; Ru—36.28; and Li—3.71. Calculated, %: Cl—50.95; N—2.52; Ru—36.31; and Li—3.75. Electronic spectrum: 203 nm (ε 2.83–104 nm), 287 nm (ε 2.08–104 nm), IR, cm^−1^: Cl-Ru-Cl 220; Ru-Cl 301; Ru-N-Ru 330; and Ru-N 1075.

### 2.2. Characterization of Complex 1

#### 2.2.1. Elemental Analysis

Elemental analysis was carried out at the Analytical Center of Institute of Problems of Chemical Physics RAS by CHNOS analyzer Vario EL cube (Elementar GmbH, Langenselbold, Germany), metals-by atomic absorption spectrometer «MGA-915» (Atompribor LLC, St. Petersburg, Russia).

#### 2.2.2. IR Spectroscopy

IR spectra were recorded on the IR Fourier spectrometer Bruker Invenio R (Bruker Optik GmbH, Ettlingen, Germany) in the range 6000÷80 cm^−1^, the sample for analysis was prepared in the tablet KBr.

#### 2.2.3. X-ray Diffraction Analysis

X-ray diffraction analysis was performed using an Agilent XCalibur CCD-diffractometer with an EOS detector at 100 K. The data were collected and processed, and the unit cell parameters were determined and refined using the CrysAlis PRO program (Agilent Technologies LTD, Yarnton, Oxfordshire, UK) [26]. In experiments, a crystal of 0.2 mm × 0.15 mm × 0.05 mm in size was used. The crystal structure was solved by the direct method followed by a series of Fourier syntheses. The positions and temperature parameters of non-hydrogen atoms were refined in the isotropic and then in the anisotropic approximation by the full-matrix least-squares method. The positions of the hydrogen atoms of a water molecule were revealed from difference syntheses and refined with restrictions on bond lengths and thermal parameters. All calculations were made using the SHELXTL 6.14 software package (Brucker, Madison, USA) [27].

#### 2.2.4. Spectrophotometric Research

Specord M-40 (Carl Zeis Industrielle Messtechnik GmbH, Oberkochen, Germany) was used to register the spectrum of the complex in a quartz cuvette (l = 1 cm).

#### 2.2.5. Cyclic Voltammetry

The cyclic voltammogram was recorded on a computerized potentiostat IPC-Compact P-8 (Elins Ltd., Chernogolovka, Russia). Cyclic voltammograms of the complexes were recorded on a glass-carbon electrode, d = 5 mm, in a sealed three-electrode cell in a nitrogen atmosphere. The auxiliary electrode was a Pt plate (1 cm^2^). All potentials given in the work were determined relative to the calomel electrode of comparison (SCE).

Potentiometric titration was carried out with the help of a compensation unit by using a polarographic analyzer EZ-7 (Laboratorni pristroje, Prague, Czech Republic). For potentiometric titration, a circuit was assembled from an indicator electrode in the sample solution and a comparison electrode. A SCE was used as a comparison electrode.

### 2.3. Kinetic Measurements

The kinetics of oxygen formation was measured on a vacuum glass device (design of the Institute of Problems of Chemical Physics of RAS). The amount of oxygen released was determined by a calibrated pressure gauge. The catalyst solution was poured into the reactor, and the cerium salt solution was poured into the side device. After preliminary degassing with freezing and thawing (3 times), the mixture was brought to the desired temperature, the solutions were mixed, and the reaction began. Using a calibrated vacuum gauge, the kinetics of oxygen evolution were monitored. At elevated temperatures, the reaction vessel was thermostated. After the reaction, gaseous products were collected in a mass spectrometer ampoule connected to the reactor. The amount of oxygen released was calculated by the formula for an ideal gas μ = PV/RT mol.

Turnover frequencies (TOFs) were defined as moles of produced product per mole of catalyst per s^−1^.

Turnover number (TONs) were defined as moles of produced product per mole of catalyst, nO_2_/n_cat_.

#### Mass Spectrometric Method of Gaseous Products Definition

The gaseous products of the water oxidation reaction were analyzed on a MI-1201B mass spectrometer (Selmi LLC, Sumy, Ukraine).

Chromato-mass spectrometric method:

Mass spectra of ruthenium complex solutions were obtained by ESI method on an LCMS-2020 liquid chromato-mass spectrometer (Shimadzu Corporation, Kyoto, Japan) equipped with a SIL-20 AC automatic sampler. Aqueous solutions were introduced into the electrospray ionization source using an automatic sampler at a flow rate of 20 μL/min. The volume of injected sample ranged from 0.1 to 3 μL. When a voltage was applied to the capillary (from 0.5 to 4.5 kV), finely dispersed sputtering of the analyzed solution occurred, the final stage of which was the field evaporation of the ions contained in the sample. Some of these ions enter the mass analyzer through a vacuum interface (*m*/*z* from 10 to 2000 are measured). As a rule, the temperature of the evaporator unit was 200 °C.

The calculated mass spectrum was obtained using the program Molecular Weight Calculator for Windows in Version 6.49 (http://www.alchemistmatt.com/, Pacific Northwest National Laboratory, Richland, USA).

## 3. Results

### 3.1. Synthesis and Properties of the Binuclear Ruthenium (IV) Nitrido Chloride Complex with Lithium Counter-Cations

For the multi-substrate concert processes, which is the OPS, the minimum distance between the transition metal ions in the catalytic center is very important. In order to obtain an effective ruthenium catalyst for the reaction of biomimetic water oxidation in PSII oxygen photosynthesis, an approach was chosen, which consisted in obtaining a ruthenium catalyst with the bridge N between the nuclei of Ru. For this purpose, a binuclear ruthenium nitrido complex Ru(IV) Li_3_(Ru_2_(μ-N)Cl_8_·(H_2_O)_2_) (**1**) was synthesized.

Red crystals of Complex **1** are highly soluble in water. The obtained X-ray diffraction data for Complex **1** correspond with the X-ray diffraction analysis of a similar potassium salt [20]. According to the X-ray diffraction data, it is a binuclear centrosymmetric complex having a linear skeleton. In the crystal structure of the complex, two ruthenium atoms are connected by a nitrogen bridge. The Ru-N distance in this complex is shorter (1.715 Å) than the Ru-O distance (1.86 Å) in its oxygen counterpart Li_4_(Ru_2_(µ-O)Cl_10_) (**2**) [23], which indicates the presence of a double bond between atoms Ru and N.

In contrast to the binuclear oxocentric Complex **2**, the behavior of which in solution has been studied by many authors [25], the solutions of nitride-centered Complex **1** have not been studied sufficiently. In particular, in [28], some characteristics of aqueous solutions of potassium salt are given (molar conductivity, electronic, and IR absorption spectra in the UV region). Information on the participation of similar complexes with both N- and O-bridges in the oxidation of water could not be found.

### 3.2. Behavior of Complex ***1*** in Dilute Aqueous Solution

**Figure 2:**
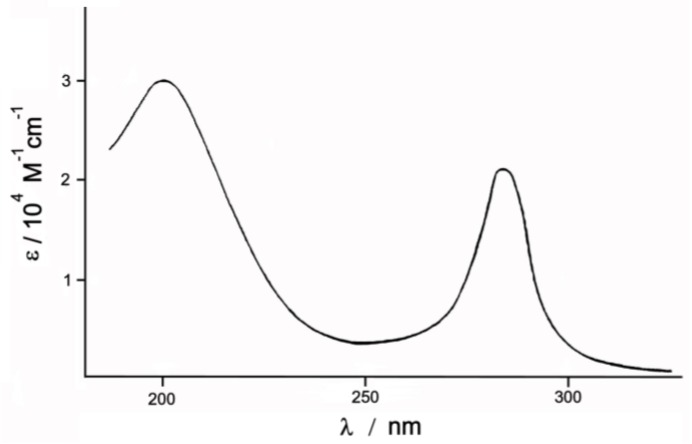
Electronic spectrum of Complex **1**, concentration 1 = 2 × 10^−3^ M.

Freshly prepared aqueous solution of the investigated Complex **1**, concentration 2 × 10^−3^ M, was painted yellow-brown. Its electronic spectrum, shown in Figure 2, contains two intensive absorption bands in the ultraviolet region with λ = 203 and 287 nm, and less intensive bands at λ = 335–380 and λ = 435 nm (not shown in Figure 2). Over time, the intensity of all the bands drops: In the first 30–60 min, a sharp drop ε in all the bands is observed, and then it slows down. A day later, only a small plateau at 410–440 nm remains in the visible area. At the same time, all the bands gradually shift (≈10 nm) towards shorter wavelengths and the solution becomes lighter. The observed gypsum shift of absorption bands in the visible region and their decreasing intensity may be a consequence of the replacement of Cl ions in the internal sphere of the complex by water molecules. This assumption is plausible, because due to the short Ru-N bond (1.715 Å) in the nitrido complex the translabilizing effect can lead to weakening of the Ru-Cl bond.

**Figure 3:**
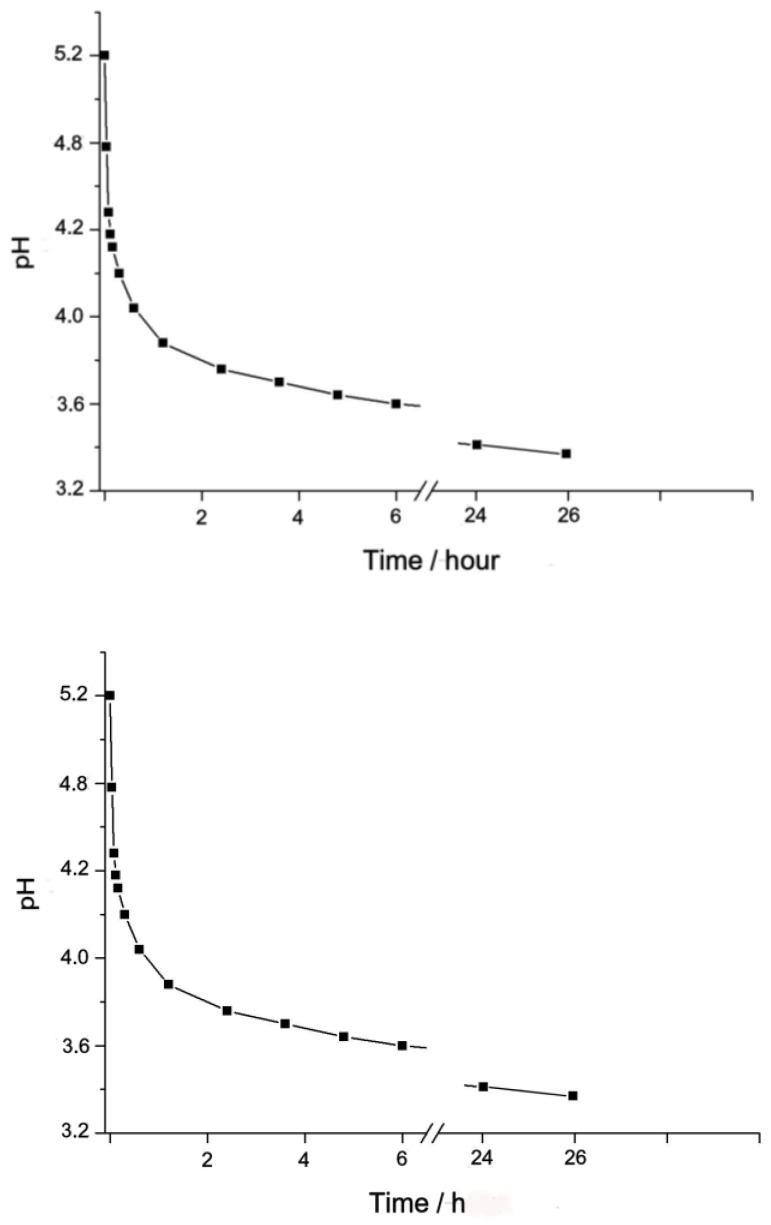
Change in pH in the aqueous solution of Complex **1** in time, concentration 1 = 2 × 10^−3^ M.

The pH value of the freshly prepared Complex **1** (2 × 10^−3^ M) is equal to 5.2 and quickly decreases during the first 40 min, as shown in Figure 3.

At the further holding of the solution, pH decreases slowly, reaching 3.85 in a day, which corresponds to the concentration of ions (H+) = 1.41 × 10^−4^ M. The increase in the concentration of H^+^ ions is due to the acid dissociation of water molecules, which replace the Cl ions in the internal coordination sphere of Complex **1**. It follows from the above that in the diluted aqueous solution, Complex **1** is stable for about one hour, and then gradually transforms into a binuclear nitrido-aqua hydroxy-chloro-complex of ruthenium, (Ru_2_^8+^(µ-N)(H_2_O)_x_(OH)_y_Cl_8-x-y_)^n−^.

### 3.3. Electrochemical Studies of Aqueous Solutions of the Complex 1

**Figure 4:**
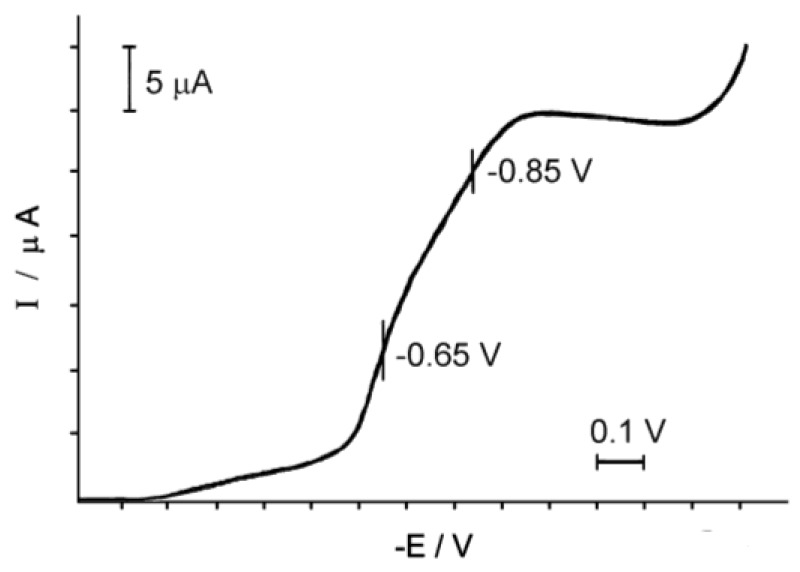
Polarogram of the aqueous solution of Complex **1,** (Ru_2_) = 2 × 10^−3^ M, the background electrolyte is 0.1 M NaCl.

The electrochemical behavior of the solution of Complex **1** is very different from the behavior of its oxygen counterpart [29]. While the binuclear Oxo-complex **2** is reduced on the platinum electrode at E = 0.40 V [23], the binuclear nitrido Complex **1** is reduced in the region of more negative potentials (Figure 4).

On the polarogram of Complex **1** in the area of negative potentials (from 0.0 to −1.3 V), two recovery waves with E = −0.65 and −0.85 V relative to our SCE are observed.

The angular coefficient of semi-logarithmic dependence, equal to the first wave of 0.06, is characteristic of the reversible single-electrode process [30], which indicates the recovery of one of the two ions Ru(IV) in the complex to Ru(III); that is, the formation of a mixed valence complex Ru_2_(IV,III). The second wave is significantly lower in height, judging by the value of the angle coefficient equal to 1.5, and is irreversible. Perhaps it responds to some other ionic form present in the solution.

**Figure 5:**
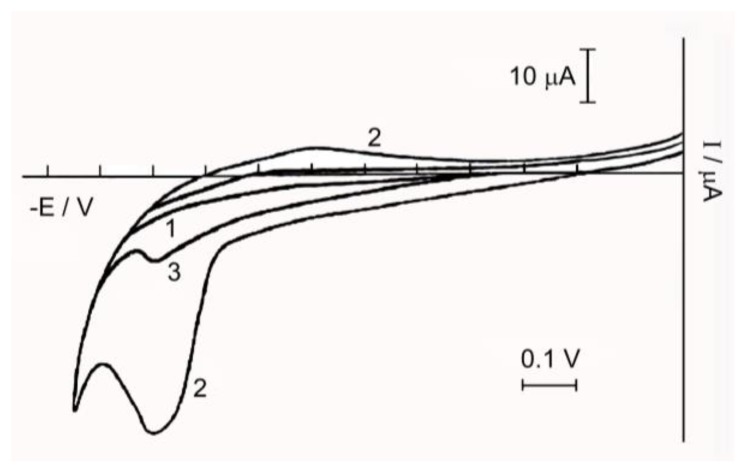
Cyclic voltammogram: Curve 1—background electrolyte 0.1 M NaCl; Curve 2—.5 × 10^−3^ M solution of Complex **1**; Curve 3—1.5 × 10^−3^ M solution of Complex **1** after oxidation on a mesh Pt-electrode.

In Figure 5 there is a cyclic voltammogram of the complex in the range from 1.0 to −0.2 V, from which it is clear that the recovery peaks of Ru(IV) are absent, and on the anode branch of the cyclic voltammogram a peak of oxidation at E = 0.95–1 V is observed, indicating the presence in the solution of Complex **1**, along with Ru(IV) and Ru(III). The height of this peak decreases after electrooxidation at E = 1.03 V on the Pt electrode, due to oxidation of Ru(III) to Ru(IV).

The anode peak at the cyclic voltammogram increases with prolonged holding of the complex solution. This is probably due to the fact that there is an intra-sphere red-ox reaction between Ru(IV) and the coordinated water molecules, leading to the accumulation of Ru(III).

All these processes start immediately after the complex dissolution in water; after an hour they slow down and continue at a fading rate of more than a day. In this case, along with the initial anions (Ru_2_^8+^(µ-N)(H_2_O)_2_Cl_8_)^3−^ complex anions of composition (Ru_2_^8+^(µ-N)(H_2_O)_x_(OH)_y_Cl_8-x-y_)^n−^ are formed in aqueous solutions.

When the pH of the solution is >6, the H_2_O molecules are completely deprotonated, and when the pH is >9, hydroxide Ru(IV) is formed.

### 3.4. The Behavior of the Complex ***1*** in Acidic Solution

**Figure 6:**
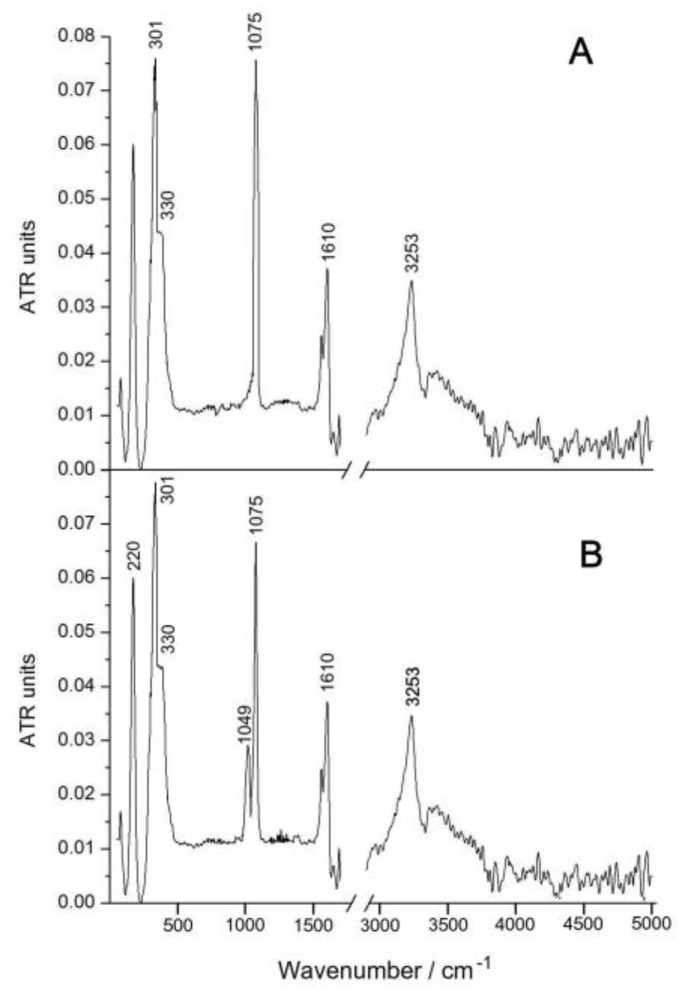
IR spectra (KBr) of the solid Complex **1** (**A**) and substance obtained after evaporation of the solution Complex **1** (**B**).

The possibility of decomposition of Complex **1** in acidic solutions is unlikely due to the short distance Ru-N. To confirm this assumption, we compared the IR spectra of the initially solid complex and the substance obtained by evaporation at room temperature in a rotary evaporator of its solution, aged for 2 days (Figure 6). The comparison of IR spectra showed that the band at 1075 cm^−1^, attributed to stretching vibrations of the Ru–N–Ru bonds, is present in the IR spectra of both substances, but the second band at 1049 cm^−1^ appears in the spectrum of the substance obtained after evaporation of the solution, which may be associated with the replacement chlorine atoms to water molecules, as indicated by some of the above methods. In the near spectral region, there are characteristic strong vibrations of the Cl–Ru–Cl bonds at 220 cm^−1^, as well as the Ru–Cl band at 301 cm^−1^ and the Ru–N shoulder at 330 cm^−1^. The stretching vibrations of the water and hydroxyl groups appear in the form of a broad band with a maximum at 3253 cm^−1^, and the deformation vibrations of adsorbed water in the form of a band at 1610 cm^−1^.

**Figure 7:**
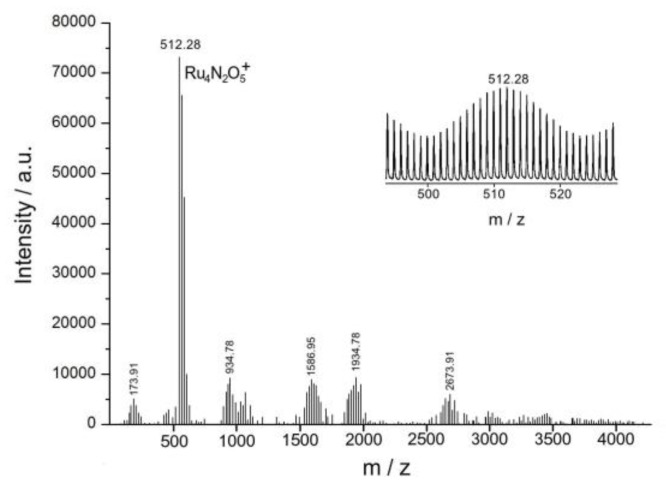
ESI mass spectrum of the product of the reduction of the initial Complex **1** (10 × 10^−3^ M in 29:70:1 CH_3_CN:H_2_O:HCOOH solvent blend).

Further research using ESI mass spectrometric analysis (Figure 7) showed that Complex **1** in an acidic environment not only preserves as the binuclear structure, but also dimerizes into a tetra-ruthenium dinitrido complex, similar to the adamantane-like Ru_4_O_6_ cluster (Richens complex), the structure of which was determined by EXAFS [31]. Each binuclear nitrido-chloride ruthenium complex Ru^IV^Ru^IV^ is two-electronically oxidant. But Complex **1** cannot oxidize water two-electronically to H_2_O_2_, because the redox potential of the Ru^IV^ Ru^IV^/Ru^III^ Ru^III^ pair (1.24 V) is much lower than the redox potential of the 2H_2_O/H_2_O_2_ pair (1.76 V), so that the water should be oxidized by a four-electron mechanism. The initial Complex **1** is a two-electron oxidant; therefore, for the four-electron oxidation of the water molecules to an oxygen molecule, two binuclear complexes should be dimerized into a tetra-nuclear cluster. The reaction stoichiometry can be written as

2(Ru^IV^ Ru^IV^) + 2 H_2_O = 2(Ru^III^ Ru^III^) + O_2_ + 4H^+^. (2)

Figure 7 shows the mass spectrum of the product of the reduction of the initial Complex **1** with water, in which the maximum peak with a mass of *m*/*z* = 512.28 corresponds to the tetra-nuclear ruthenium cation Ru_4_N_2_O_5_^+^ and its isotopic distribution.

This tetra-nuclear ruthenium complex during the oxidation of water by Ce (IV) compounds in APS also catalyzes the four-electron oxidation of water by Reaction (3).

4Ce^4+^ + 2H_2_O = 4 Ce^3+^ + O_2_ + 4H^+^. (3)

In the catalytic process for the regeneration of (Ru^III^ Ru^III^)_2_ to its active state (Ru^IV^ Ru^IV^)_2_, it is necessary to consume four one-electron oxidizers of Ce (IV).

Of course, the collision of four cerium molecules does not occur simultaneously; sequential bimolecular reactions of stepwise oxidation of the reduced complex [Ru^III^ Ru^III^]_2_ to [Ru^IV^ Ru^IV^]_2_ with its subsequent decomposition into the reduced catalyst, O_2_ and 4H^+^ by Reaction (2), take place.

### 3.5. Kinetic Measurements of Reaction of Water Oxidation with Exogenous Oxidizer

**Figure 8:**
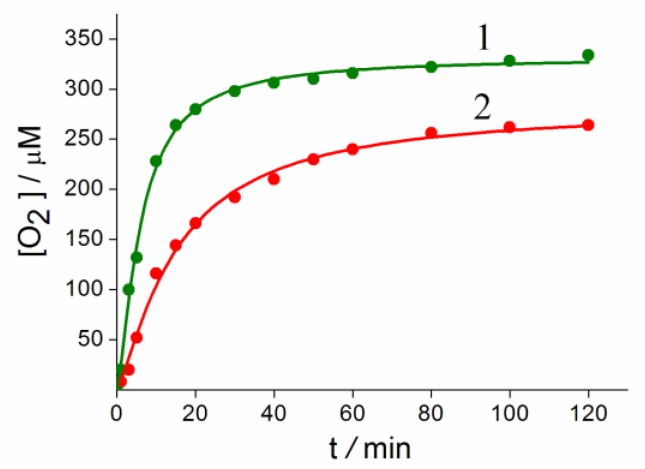
Kinetics of O_2_ formation at water oxidation by Ce(IV) compounds catalyzed by Complexes **1** (1) and **2** (2).

The possibility of decomposition of the complex in acid solutions is unlikely due to the short distance of Ru-N. In this connection, the reaction of water oxidation with exogenous oxidizer (NH_4_)_2_Ce(NO_3_)_6_ (Ce(IV)), catalyzed by Complex **1**, was studied and compared with the similar Complex **2** [23]. Counter-catione Li^+^ in Complex **1** has a significant impact on the stability and activity of the catalyst. Kinetic curves of oxygen formation in 2.5 M HCl during biomimetic oxidation of water by an excess of Ce(IV) compounds in coordination Spheres of **1** and **2** differ (Figure 8).

Conditions: **1** and **2** = 1.1 × 10^−6^ mole, CAN = 1.1 × 10^−4^ mole, 22 °C, 2.5 M HCl.

TOF **1** = 0.33 s^−1^, TON = 304

TOF **2** = 0.12 s^−1^, TON = 240.

**Figure 9:**
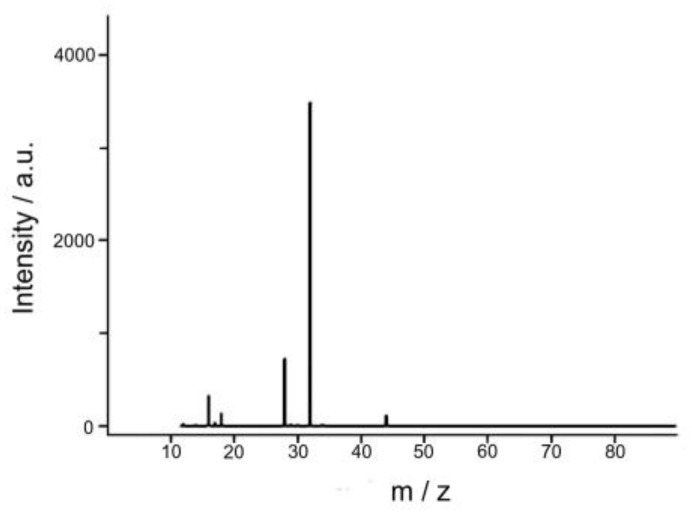
Mass spectrum of the gas phase products of the water oxidation reaction by Ce(IV) compounds catalyzed by Complex **1**.

Analysis of the gas phase products of the reaction of water oxidation by mass spectrometric method showed that the only one product of the reaction is O_2_ (*m/z* = 32) (Figure 9).

From Figure 8 it can be clearly seen that Complex **1** is more active (TOF 0.33 s^−1^, TON 304) compared with Complex **2** (TOF = 0.12 s^−1^, TON 240). The rather high activity **1** indicates the strength of the catalyst structure in comparison with other previously described systems containing organic ligands and the labile bonds Ru-O-Ru [6,7,8,9]. The effect of the Li^+^ cation is not yet clear, and it is the subject of further research.

## 4. Conclusions

In this paper, important experimental results on the synthesis and physico-chemical properties of an inorganic catalyst of biomimetic water oxidation for the APS and its use as a precursor in an acidic medium were obtained. Thus, the preparation of a lithium salt of the binuclear µ-nitrido-chloride complex has opened the possibility of using it as a precursor in the oxidation of water in APS. In an acidic environment, the complex self-organizes into a tetra-nuclear cluster, which is a catalyst for the four-electron oxidation of water to form an O_2_ molecule. The activity of the catalyst is ~3 times higher than that of a similar complex with oxygen bridges.
